# Psychosocial Disadvantage During Childhood and Midlife Health: NIMHD Social Epigenomics Program

**DOI:** 10.1001/jamanetworkopen.2024.21841

**Published:** 2024-07-29

**Authors:** Ryan L. Brown, Katie E. Alegria, Elissa Hamlat, A. Janet Tomiyama, Barbara Laraia, Eileen M. Crimmins, Terrie E. Moffitt, Elissa S. Epel

**Affiliations:** 1 Center for Health and Community, University of California, San Francisco; 2 Department of Psychology, University of California, Los Angeles; 3 School of Public Health, University of California, Berkeley; 4 Leonard Davis School of Gerontology, University of Southern California, Los Angeles; 5 Department of Psychology and Neuroscience, Duke University, Durham, North Carolina

## Abstract

**Importance:**

Low childhood socioeconomic status (SES) is a social hallmark of aging that contributes to adult health disparities and earlier morbidity and mortality. Childhood perceptions of stress are associated with child health outcomes and may contribute to premature biological aging into adulthood.

**Objective:**

To describe the association of childhood SES and perceived stress with midlife insulin resistance and epigenetic age and to explore whether late adolescent adiposity mediates the observed associations.

**Design, Setting, and Participants:**

The longitudinal cohort National Heart, Lung, and Blood Institute Growth and Health Study enrolled girls aged 10 years from January 1987 to May 1988, and followed them up to 19 years of age. Participants from Richmond, California, were recruited again at midlife in 2016 to assess insulin resistance and epigenetic age. Analyses were conducted from August 2, 2023, to March 18, 2024. A total of 433 participants were eligible and included in the analyses (specific sample sizes ranged across analyses from 303 to 391).

**Exposures:**

Childhood levels of SES at 10 years of age (parental educational level and income) and perceived stress at 11 years of age.

**Main Outcomes and Measures:**

The hypotheses tested were formulated after data collection. Outcomes included the homeostatic model assessment of insulin resistance (HOMA-IR) and the GrimAge and DunedinPACE epigenetic clocks. Waist circumference in late adolescence was tested as a mediator.

**Results:**

Among the 433 participants, the mean (SD) age was 39.4 (1.2) years; 218 (50.3%) were Black and 215 (49.7%) were White; and 135 (31.2%) had parents with a college degree or higher. Higher parental educational level was associated with lower HOMA-IR (B = −0.22 [95% CI, −0.41 to −0.02]; *P* = .03), lower midlife GrimAge (B = −1.76 [95% CI, −2.85 to −0.66] years; *P* = .002), and slower midlife DunedinPACE (B = −0.03 [95% CI, −6.29 to −0.002]; *P* = .04). Childhood perceived stress was indirectly associated through late adolescent adiposity with midlife HOMA-IR (B = 0.01 [95% CI, 0.001-0.01]; *P* = .02) and midlife GrimAge (B = 0.02 [95% CI, 0.003-0.04] years; *P* = .01).

**Conclusions and Relevance:**

In this longitudinal cohort study of midlife health and aging, childhood social hallmarks of aging were associated with midlife insulin resistance and epigenetic age (GrimAge and DunedinPACE). Future studies should identify malleable factors that may slow the impact of social hallmarks of aging.

## Key Points

**Question:**

Are childhood social hallmarks of aging (socioeconomic status and stress) associated with adult insulin resistance and epigenetic age?

**Findings:**

In the National Heart, Lung, and Blood Institute Growth and Health Study, a longitudinal cohort study tracking 433 girls from 10 to 40 years of age, lower parental educational level and higher childhood perceived stress were associated with greater midlife risk (higher insulin resistance and higher epigenetic age) similarly across Black and White women.

**Meaning:**

These results suggest that childhood structural and psychosocial factors are associated with aging-related health disparities.

## Teaser

This cohort study examines independent and additive associations of low childhood socioeconomic status and perceived stress in childhood with insulin resistance and epigenetic aging among women followed up from 10 to 40 years of age.

## Introduction

Psychosocial disadvantage in childhood contributes to health disparities in aging-related outcomes through biological and behavioral pathways.^1,2,3,4,5^ In adults, the social hallmarks of aging (eg, low socioeconomic status [SES] and high psychological stress) explain more variance in multimorbidity from midadulthood to late adulthood compared with traditional biological hallmarks of aging (eg, telomere attrition and genomic instability).^6^ Most studies have focused on adulthood and/or retrospective measures of SES or objective indicators of childhood hardship, which may contribute to higher subjective appraisals of stress but are not a direct measure of psychological stress. In this study, we leveraged the 30-year longitudinal National Heart, Lung, and Blood Institute Growth and Health Study (NGHS) to examine independent and additive associations of low childhood SES and perceived stress in childhood with 2 well-established factors associated with mortality: insulin resistance^7,8^ and epigenetic aging.^9^

Socioeconomic disadvantage during childhood confers health risks by limiting access to educational opportunities and health care, which can translate into continued socioeconomic disadvantage and limited access to health care into adulthood.^10^ Individuals with low SES can also incur social stress, and a sense of lack of safety in the world can promote disease through worsened health behaviors and greater dysregulation of stress-related regulatory systems.^11^ Childhood socioeconomic disadvantage is associated with cardiometabolic health at various points across one’s life.^12,13,14^ In a study of more than 3000 people (aged 35-45 years), lower levels of parental education were associated with worse adult metabolic functioning (ie, levels of insulin, glucose, cholesterol, and triglycerides and waist circumference).^15^ There are robust associations between adverse childhood experiences and insulin resistance in adulthood.^16,17^ However, these assessments are typically measured retrospectively and cannot capture appraisals of stress, which may be important for understanding one’s susceptibility to adversity in their social environments.^18^

Psychosocial disadvantage in childhood may promote aging-related disease risk through accelerated epigenetic aging. Second-generation epigenetic clocks (eg, GrimAge)^19^ were trained on phenotypic biomarkers of aging and may represent aging pathways more proximal to social adversity.^20^ Among current epigenetic clocks, GrimAge is the best estimator of lifespan and health span.^9,21,22,23^ In the Irish Longitudinal Study on Aging,^24^ childhood poverty was associated with 2 additional years of epigenetic age assessed with GrimAge, which supports evidence from other studies finding childhood socioeconomic disadvantage associated with accelerated GrimAge.^25,26^ However, not all studies have found a link between early-life SES and epigenetic age acceleration.^27^ Further, DunedinPACE, a newer pace of aging measure, was trained on a longitudinal sample of adults in midlife, making it uniquely suited for the purposes of the present study. DunedinPACE has been associated with parental and adult SES^28,29^ and objective, retrospective measures of childhood stress (eg, adverse childhood experiences).^30^

Plausible physiological pathways may help explain how early psychosocial stress contributes to cardiometabolic risk and epigenetic age. Long-term exposure to psychological stress can contribute to greater visceral fat in adults and children,^31,32,33,34^ which can then decrease the effectiveness of insulin and the uptake of glucose, putting one at risk of a prediabetic state.^35^ Central adiposity, as measured by waist circumference, is associated with insulin resistance in childhood^36,37^ and older adulthood.^38^ Even when accounting for body mass index and body fat percentage, waist circumference remained associated with insulin sensitivity in a population of adults aged 50 to 95 years.^38^ In a study of 34 710 European adults, higher waist circumference was identified as a causal risk factor for accelerated epigenetic age,^39^ adding to the existing literature on waist circumference and epigenetic age.^40^ To our knowledge, there are no existing longitudinal studies examining waist circumference as a mediator between childhood stress and accelerated biological age.

We hypothesized that lower childhood SES and higher levels of childhood perceived stress would each be associated with elevated insulin resistance, more advanced epigenetic age (GrimAge), and faster epigenetic aging (DunedinPACE) at midlife. Perceived stress may alter the observed associations between childhood SES and these outcomes. Thus, we explored whether there were independent or additive associations among SES, perceived stress, and our outcomes. We also explored whether waist circumference would mediate the associations between SES and perceived stress and the outcome variables.

## Methods

### Procedure

Data originated from NGHS, a longitudinal cohort study that annually assessed health-related outcomes among Black and White girls throughout childhood, with enrollment beginning in January 1987 and continuing through May 1988 (median age, 10 through 19 years). Recruitment and eligibility of the original study were described in detail previously.^41^ A one-time follow-up point in 2016 again recruited participants from Richmond, California, in midlife (40 years of age). The primary analyses in this report pull from the first time the original study included an assessment of childhood SES (10 years of age) and childhood perceived stress (11 years of age), as well as the last wave of original data collection for late adolescent adiposity (19 years of age), and the follow-up point for adult health and aging (40 years of age) (see eFigure 1 in Supplement 1 for the study timeline and eTable 1 in Supplement 1 for comparison of full, analytic, and excluded samples).

The exclusion criteria for the follow-up study consisted of pregnancy within 3 months of recruitment and living abroad, institutionalized, or incarcerated. Recruitment strategies included emails, telephone calls, and social media use. Eligible participants provided written informed consent and participated in (1) a survey; (2) a home or clinic visit, including measurement of waist circumference and saliva collection; and (3) other biospecimen collection. We followed the Strengthening the Reporting of Observational Studies in Epidemiology (STROBE) reporting guideline for cohort studies. The study was approved by the Institutional Review Board of the University of California, Berkeley.

### Measures

#### Childhood and Late Adolescent Measures

Childhood SES at 10 years of age was measured by (1) annual household income in US dollars (<$10 000, $10 000-$19 999, $20 000-$39 999, and ≥$40 000) and (2) maximum parental educational level (high school or lower, some college, and college degree or higher). Baseline SES (10 years of age) was reported by participants’ mothers. Perceived stress at 11 years of age was assessed by the 14-item Perceived Stress Scale (score range, 0-56; higher scores indicate higher levels of perceived stress), which measures psychological appraisals of stress experienced within the past month.^42^ Waist circumference at 11 and 19 years of age was obtained through above-waist measurements by trained staff members during home visits.^43^

#### Midlife Study Measures

The second-generation GrimAge epigenetic clock^19^ used DNA methylation assessed in saliva sampled at 40 years of age and assayed at the UCLA Neuroscience Genomics Core (UNGC; Horvath laboratory) using a commercially available array (Infinium HumanMethulation450 BeadChip; Illumina, Inc). GrimAge, version 2, uses age, being female, and 10 DNA methylation–based biomarkers.^19^

The DunedinPACE measure of pace of aging was assessed in saliva at 40 years of age by the UNGC laboratory. Values greater than 1 indicate a faster pace of aging.^44^ The homeostatic model assessment of insulin resistance (HOMA-IR) was calculated from blood draws at 40 years of age assessing fasting glucose and insulin concentrations using the following standard formula:

Glucose Level (in mg/dL) × Insulin Level (in μU/mL)/405

Each participant’s educational level was self-reported at 40 years of age (high school or lower, some college, or college degree or higher).

#### Covariates

Race (Black and White) was both self-reported and identified by each girl’s parent or guardian at baseline. Age was self-reported at the midlife point. Smoking status (current smoker or nonsmoker) was self-reported at 14 and 40 years of age. Diabetes status (yes or no) was self-reported as a diagnosis at 14 and 40 years of age. The yes category includes prediabetes.

### Statistical Analysis

All data analyses were completed in R, version 4.2.2, using R Studio (R Project for Statistical Computing) from August 2, 2023, to March 18, 2024. HOMA-IR was natural log transformed before all analyses to better approximate a normal distribution. Statistical significance was defined as 2-sided *P* < .05. We removed 13 outliers (ie, >2 SD) for HOMA-IR (outlier range, 9.8-33.7) from the final analytic sample. Individual associations were examined before and after including plausible mediators: diabetes status, smoking status, and estimated cell type composition (for epigenetic clock analyses in line with recommendations for standardized reporting).^45,46^ In the remaining models, HOMA-IR models included covariates of age, diabetes status, smoking status, and race. Epigenetic clock analyses included these covariates as well as analysis cluster and estimated cell type composition to account for confounding from cell composition. Thus, GrimAge and DunedinPACE models include counts of naive CD8^+^ and CD4^+^ T cells, exhausted cytotoxic CD8^+^ T cells (CD8^+^/CD28^–^/CD45^–^), natural killer cells, granulocytes, monocytes, B cells, and CD4^+^ and CD8^+^ T cells.^47^

For each aim, multivariable linear regression models were estimated. Hierarchical multivariable regressions were estimated along with model comparisons to determine whether adding a block significantly improved the fit of each model. We generated η_p_^2^ effect sizes. These analyses had 3 blocks of variables based on findings in the prior step: (1) covariates only (all outcomes: diabetes status, smoking status, race; GrimAge and DunedinPACE: analysis cluster, estimated cell type compositions), (2) covariates plus SES, and (3) covariates plus SES plus childhood perceived stress. The mediation R package was used for all exploratory mediation analyses to compute unstandardized indirect associations with 1000 bootstrapped samples.^48^

## Results

The 433 participants all identified as women; 218 (50.3%) were Black and 215 (49.7%) were White. The mean (SD) age at follow-up was 39.4 (1.2) years (range, 36-43 years), 135 participants (31.2%) had parents with a college degree or higher, and participants reported a mean score of 25.69 (6.68) on the Perceived Stress Scale (indicating relatively high levels of childhood perceived stress). Table 1 provides demographic characteristics and eFigure 2 in Supplement 1 provides correlations. Broadly, the results described herein do not differ across the Black and White women in our sample (eResults 1 in Supplement 1).

**Table 1.** Participant Characteristics

Characteristic	Values (N = 433)^a^
Age, mean (SD) [range], y	39.4 (1.2) [36-43]
Race	
Black	218 (50.3)
White	215 (49.7)
Childhood parental income, US$	
<10 000	80 (18.5)
10 000-19 999	74 (17.1)
20 000-39 999	122 (28.2)
≥40 000	157 (36.3)
Parental educational level	
High school or lower	98 (22.6)
Some college	200 (46.2)
College degree or higher	135 (31.2)
Current smoking status	
No	355 (82.0)
Yes	78 (18.0)
Current diabetes status^b^	
No	370 (86.2)
Yes	59 (13.8)
Late adolescent waist circumference, mean (SD) [range], cm	76.31 (12.80) [58.50-127.05]
Childhood Perceived Stress Scale score, mean (SD) [range]^c^	25.69 (6.68) [8-50]
Midlife HOMA-IR, median (range)^d^^,^^e^	1.75 (0.44-9.28)
Midlife GrimAge2, mean (SD) [range], y^e^	61.60 (5.59) [47.35-82.04]
Midlife DunedinPACE, mean (SD) [range]^e^	1.28 (0.17) [0.95-1.62]

Abbreviation: HOMA-IR, homeostatic model assessment of insulin resistance.

^a^ Unless otherwise indicated, data are expressed as No. (%) of participants. Percentages have been rounded and may not total 100.

^b^ Missing for 4 participants.

^c^ Measured at 11 years of age. Scores range from 0 to 56, with higher scores indicating higher levels of perceived stress.

^d^ Raw variable presented here; log-transformed variable was used in analyses. Outliers were removed to accurately represent the analytic sample. The median is presented due to nonnormal distribution.

^e^ Measured at 40 years of age.

### Individual Associations of Childhood Psychosocial Disadvantage on Midlife Insulin Resistance and Epigenetic Age

#### Childhood SES

##### Parental Education

Having parents with a college degree or higher was associated with lower midlife HOMA-IR levels (B = −0.22 [95% CI, −0.41 to −0.02]; *P* = .03), compared with those whose parents had a high school degree or lower. Having parents with some college (B = −1.56 [95% CI, −2.82 to −0.30] years; *P* = .02) or with a college degree (B = −2.57 [95% CI, −3.96 to −1.18] years; *P* < .001) was associated with lower midlife GrimAge compared with those whose parents had a high school degree or lower. There were no associations between parental educational level and midlife DunedinPACE.

After adjusting for midlife diabetes, smoking status, and estimated cell type composition (GrimAge and DunedinPACE only), having parents with a college degree or higher was associated with lower midlife HOMA-IR (B = −0.22 [95% CI, −0.41 to −0.02]; *P* = .03), compared with those whose parents had a high school degree or lower. Having parents with some college (B = −1.08 [95% CI, −2.06 to −0.09] years; *P* = .03) or a college degree (B = −1.76 [95% CI, −2.85 to −0.66] years; *P* = .002) was associated with lower midlife GrimAge compared with those whose parents had a high school degree or lower. Having parents with a college degree or higher was associated with a slower midlife DunedinPACE (B = −0.03 [95% CI, −6.29 to −0.002]; *P* = .04) compared with those whose parents had a high school degree or lower.

##### Parental Income

There was no association between childhood parental income and HOMA-IR. Having a parental income of $20 000 to $39 999 (B = −1.84 [95% CI, −3.34 to −0.35] years; *P* = .02) or at least $40 000 (B = −2.13 [95% CI, −3.62 to −0.65] years; *P* = .005) was associated with lower midlife GrimAge, and having a parental income of at least $40 000 was associated with slower midlife DunedinPACE (B = −0.05 [95% CI, −1.00 to −0.01]; *P* = .03) compared to those with a parental income of less than $10 000. There were no other associations between childhood parental income and midlife DunedinPACE.

After adjusting for midlife diabetes, smoking status, and estimated cell type composition (GrimAge and DunedinPACE only), there were no associations between childhood parental income and midlife HOMA-IR. Having a parental income of $10 000 to $19 999 was associated with higher midlife GrimAge (B = 1.33 [95% CI, 0.07-2.58]; *P* = .04) compared with those who had a parental income of less than $10 000. There were no associations between childhood parental income and midlife DunedinPACE.

#### Childhood Perceived Stress

Higher childhood perceived stress was associated with higher midlife HOMA-IR (B = 0.017 [95% CI, 0.01-0.03]; *P* = .001). Childhood perceived stress was not associated with midlife GrimAge (B = 0.04 [95% CI, −0.03 to 0.12] years; *P* = .24) or with midlife DunedinPACE (B = 0.001 [95% CI, −0.00 to 0.003]; *P* = .68).

After adjusting for midlife diabetes, smoking status, and estimated cell type composition (GrimAge and DunedinPACE only), higher childhood perceived stress remained associated with higher midlife HOMA-IR (B = 0.02 [95% CI, 0.01-0.03]; *P* = .004). Childhood perceived stress was not associated with midlife GrimAge (B = 0.02 [95% CI, −0.04 to 0.08] years; *P* = .58) or midlife DunedinPACE (B = −0. 00002 [95% CI, −0.002 to 0.002]; *P* = .98).

### Additive Associations of Childhood Psychosocial Disadvantage on Midlife Insulin Resistance and Epigenetic Age

#### HOMA-IR

Having parents with a college degree or higher (vs a high school degree or lower) was associated with lower midlife HOMA-IR (standardized β = −0.37 [95% CI, −0.66 to −0.08]; *P* = .01). Reporting higher childhood perceived stress was also associated with higher midlife HOMA-IR (standardized β = 0.14 [95% CI, 0.04-0.25]; *P* = .006) (Table 2).

**Table 2.** Independent and Additive Associations of Stress at 11 Years of Age and Childhood Parental SES With Insulin Resistance at 40 Years of Age From 336 Observations

Factor	Covariate model		SES model		Combined model		
	Standardized β coefficient (95% CI)^a^	*P* value	Standardized β coefficient (95% CI)^b^	*P* value	Standardized β coefficient (95% CI)^c^	*P* value	η_p_^2^ Effect size
Intercept	−0.28 (−0.43 to −0.131)	.54	−0.06 (−0.32 to 0.20)	.53	−0.03 (−0.29 to 0.23)	.55	NA
Age	−0.01 (−0.11 to 0.091)	.85	−0.004 (−0.11 to 0.10)	.94	−0.02 (−0.12 to 0.08)	.73	.00003
Diabetes^d^	0.87 (0.54 to 1.201)	<.001	0.86 (0.54 to 1.19)	<.001	0.79 (0.47 to 1.12)	<.001	.09
Current smoker^d^	0.05 (−0.22 to 0.31)	.73	0.01 (−0.26 to 0.28)	.97	−0.02 (−0.29 to 0.25)	.89	.001
Race^e^	0.36 (0.16 to 0.57)	.001	0.31 (0.10 to 0.52)	.004	0.32 (0.11 to 0.53)	.003	.04
Parental educational level							
High school or lower	NA	NA	1 [Reference]	NA	1 [Reference]	NA	.02
Some college	NA	NA	−0.18 (−0.44 to 0.08)	.18	−0.20 (−0.46 to 0.06)	.13	
College degree or higher	NA	NA	−0.33 (−0.62 to −0.04)	.03	−0.37 (−0.66 to −0.08)	.01	
Childhood perceived stress	NA	NA	NA	NA	0.14 (0.04 to 0.25)	.006	.02

Abbreviations: NA, not applicable; SES, socioeconomic status.

^a^ *R*^2^ = 0.116; adjusted *R*^2^ = 0.106.

^b^ *R*^2^ = 0.129; adjusted *R*^2^ = 0.113. *P* = .08 compared with covariate model.

^c^ *R*^2^ = 0.149; adjusted *R*^2^ = 0.131. *P* = .006 compared with covariate model.

^d^ A value of 1 indicates yes; a value of 0, no.

^e^ A value of 1 indicates White; a value of 2, Black.

#### GrimAge

Having parents with some college (standardized β = −0.19 [95% CI, −0.37 to −0.01]; *P* = .04) or a college degree or higher (standardized β = −0.26 [95% CI, −0.47 to −0.05]; *P* = .01) was associated with lower GrimAge compared with those whose parents had a high school degree or lower. A parental income of $10 000 to $19 999 was associated with higher midlife GrimAge (standardized β = 0.26 [95% CI, 0.03-0.48]; *P* = .03) compared with those who had a parental income of less than $10 000 (Table 3).

**Table 3.** Independent and Additive Associations of Stress at 11 Years of Age and Childhood Parental SES on Epigenetic Age at 40 Years of Age From 391 Observations

Predictors	Covariate model		SES model		Combined model		
	Standardized β coefficient (95% CI)^a^	*P* value	Standardized β coefficient (95% CI)^b^	*P* value	Standardized β coefficient (95% CI)^c^	*P* value	η_p_^2^ Effect size
(Intercept)	−0.39 (−0.83 to 0.06)	.65	−0.19 (−0.66 to 0.29)	.84	−0.18 (−0.65 to 0.30)	.81	NA
Age	0.08 (0.003 to 0.15)	.04	0.07 (0.01 to 0.14)	.04	0.07 (−0.002 to 0.14)	.06	.03
Diabetes^d^	0.11 (0.04 to 0.18)	.003	0.10 (0.03 to 0.17)	.005	0.09 (0.03 to 0.16)	.008	.02
Current smoker^d^	0.98 (0.80 to 1.17)	<.001	0.91 (0.72 to 1.10)	<.001	0.90 (0.71 to 1.09)	<.001	.27
Race^e^	0.47 (0.32 to 0.61)	<.001	0.41 (0.25 to 0.57)	<.001	0.42 (0.26 to 0.57)	<.001	.07
Cluster	−0.02 (−0.47 to 0.43)	.94	−0.03 (−0.47 to 0.41)	.91	−0.04 (−0.48 to 0.40)	.87	.23
Cytotoxic T cells	−0.02 (−0.10 to 0.06)	.57	−0.02 (−0.10 to 0.06)	.67	−0.02 (−0.10 to 0.06)	.62	.05
Naive CD8^+^ T cells	−0.13 (−0.22 to −0.05)	.003	−0.14 (−0.23 to −0.06)	.001	−0.15 (−0.23 to −0.06)	.001	.12
Naive CD4+ T cells	0.13 (0.06 to 0.21)	.001	0.13 (0.05 to 0.20)	.001	0.12 (0.05 to 0.20)	.001	.08
Granulocytes	0.88 (−0.12 to 1.88)	.08	1.07 (0.08 to 2.05)	.03	1.07 (0.08 to 2.05)	.03	.16
Monocytes	0.47 (−0.00 to 0.94)	.05	0.52 (0.06 to 0.99)	.03	0.51 (0.05 to 0.98)	.03	.004
B cells	−0.25 (−0.52 to 0.01)	.06	−0.21 (−0.48 to 0.05)	.11	−0.20 (−0.46 to 0.06)	.14	.01
CD4^+^ T cells	0.97 (0.40 to 1.54)	.001	1.07 (0.50 to 1.63)	<.001	1.06 (0.50 to 1.62)	<.001	.03
CD8^+^ T cells	0.05 (−0.07 to 0.17)	.42	0.05 (−0.07 to 0.17)	.42	0.05 (−0.08 to 0.17)	.47	.001
Natural killer cells	0.14 (0.002 to 0.27)	.05	0.14 (0.002 to 0.27)	.05	0.14 (0.004 to 0.27)	.04	.01
Parental educational level							
High school or lower	NA	NA	1 [Reference]	NA	1 [Reference]	NA	.03
Some college	NA	NA	−0.17 (−0.35 to 0.01)	.06	−0.19 (−0.37 to −0.01)	.04	
College degree or higher	NA	NA	−0.25 (−0.45 to −0.04)	.02	−0.26 (−0.47 to −0.05)	.01	
Childhood parental income, US$							
10 000	NA	NA	1 [Reference]	NA	1 [Reference]	NA	.03
10 000-19 999	NA	NA	0.25 (0.02 to 0.47)	.03	0.26 (0.03 to 0.48)	.03	
20 000-39 999	NA	NA	−0.10 (−0.31 to 0.11)	.35	−0.09 (−0.30 to 0.12)	.41	
≥40 000	NA	NA	−0.02 (−0.24 to 0.19)	.83	−0.02 (−0.23 to 0.20)	.89	
Childhood perceived stress	NA	NA	NA	NA	0.04 (−0.03 to 0.11)	.27	.003

Abbreviations: NA, not applicable; SES, socioeconomic status.

^a^ *R*^2^ = 0.555; adjusted *R*^2^ = 0.539.

^b^ *R*^2^ = 0.579; adjusted *R*^2^ = 0.558. *P* < .001 compared with covariate model.

^c^ *R*^2^ = 0.581; adjusted *R*^2^ = 0.558. *P* = .27 compared with covariate model.

^d^ A value of 1 indicates yes; a value of 0, no.

^e^ A value of 1 indicates White; a value of 2, Black.

In supplemental analyses, we examined whether there were any interactions between these variables and observed limited evidence. Results are found in eTables 2 to 5 in Supplement 1.

### Adiposity in Late Adolescence as a Mediator of the Association Between Childhood Psychological Stress and Adult Health

#### HOMA-IR

Higher childhood perceived stress was associated with higher HOMA-IR at 40 years of age (B = 0.01 [95% CI, 0.002-0.02]; *P* = .02) (n = 303). In path A, childhood perceived stress was associated with higher adiposity in late adolescence (B = 0.31 [95% CI, 0.10-0.53]; *P* = .005); in turn, in path B, higher adiposity in late adolescent was associated with elevated midlife HOMA-IR (B = 0.02 [95% CI, 0.01-0.02]; *P* < .001). Childhood perceived stress was associated with HOMA-IR at 40 years of age (B = 0.01; [95% CI, 0.001-0.02]; *P* = .03), but the association was not direct (B = 0.01 [95% CI, −0.003 to 0.02]; *P* = .20). Instead, childhood perceived stress was associated through adiposity in late adolescence with midlife HOMA-IR (B = 0.01 [95% CI, 0.001-0.01]; *P* = .02).

#### GrimAge

Higher childhood perceived stress was not associated with higher midlife GrimAge, though the estimate remained in the hypothesized direction (standardized β = 0.05 [95% CI, −0.01 to 0.11]; *P* = .11) in path C (n = 346). In path A, consistent with HOMA-IR, higher childhood perceived stress was associated with higher adiposity in late adolescence (standardized β = 0.28 [95% CI, 0.08-0.48]; *P* = .006); in turn, in path B, higher adiposity in late adolescence was associated with higher midlife GrimAge (standardized β = 0.06 [95% CI, 0.02-0.09]; *P* < .001). There was not a total association of childhood perceived stress and midlife GrimAge (standardized β = 0.05 [95% CI, −0.01 to 0.12]; *P* = .11), nor was there a direct association (standardized β = 0.03 [95% CI, −0.03 to 0.09]; *P* = .22). Instead, childhood perceived stress was indirectly associated through adiposity in late adolescence with GrimAge (standardized β = 0.02 [95% CI, 0.003-0.04]; *P* = .01) (sensitivity analyses are provided in eResults 2 and 3 in Supplement 1).

### Intergenerational Educational Experiences and Midlife Insulin Resistance and Epigenetic Age

Exploratory post hoc analyses identified that parental educational level was indirectly associated with GrimAge through one’s own level of education. Findings of the post hoc analyses are provided in eResults 4 in Supplement 1.

## Discussion

This longitudinal cohort study examined associations between social psychological disadvantage measured in childhood (lower SES and higher perceived stress) and age-related health in midlife (insulin resistance and epigenetic age) in a sample of Black and White women. Having parents with a lower level of education was associated with elevated insulin resistance, higher GrimAge, and faster midlife DunedinPACE; these findings remained significant even after controlling for midlife diabetes and smoking status. Higher childhood perceived stress was associated with elevated insulin resistance. Childhood perceived stress was also indirectly associated with midlife insulin resistance and GrimAge through late adolescent abdominal adiposity. These findings emphasize the importance of early childhood psychosocial disadvantage in shaping long-term health.

Having higher perceived stress as a child and a parent who had a lower level of education were uniquely and independently associated with elevated insulin resistance in midlife. This finding complements previous research showing lower parental educational levels were associated with baseline levels of childhood insulin resistance and change in insulin resistance over 3 years.^14^ Herein, this association persists into midadulthood. Moreover, we found a potential pathway for the associations of childhood disadvantage on adult health, such that adiposity in late adolescence mediated the association between childhood perceived stress and insulin resistance and GrimAge. This finding highlights preliminary evidence for a lifespan perspective on psychosocial stress, adiposity, and age-related health, which would be enhanced by examining these individual factors in the context of broader structural systems (eg, schools).

When considering epigenetic age, parental educational level and parental income emerged as associated with midlife GrimAge. Exploratory analyses highlighted that parental educational level was associated with midlife GrimAge through one’s own educational level, possibly reflecting intergenerational benefits of higher educational experiences. A previous study in this cohort^26^ found similar results that education but not income was related to epigenetic age. Surprisingly few of the indicators of psychosocial disadvantage were associated with the pace of aging as measured by DunedinPACE, despite other studies finding that lower SES relates to faster pace of aging.^28,49^ Differing indicators of childhood stress, specifically measures of early-life adversity, have been associated with a faster pace of aging in midlife, but we did not find an association between childhood perceived stress and DunedinPACE.^30,50^ Future work should investigate these and other facets of SES to better understand area-level effects (eg, familial, neighborhood, region) for tailoring social interventions.^51^

### Strengths and Limitations

There are several important strengths to this study. We followed up women over 30 years, assessing appraisals of stress and SES during childhood rather than retrospectively. These are some of the highest-quality data available to test a life course perspective on stress, adiposity, and midlife health that reflect a highly plausible mechanism (adiposity) through which early-life stress (whether structural or psychosocial) can contribute to health as one ages.

This study also has some limitations. We cannot conclude causality between childhood psychosocial disadvantage and adult health, given the longitudinal observational nature of this study, nor can we fully disentangle the associations among childhood SES, psychosocial stress, and adiposity with this design, given the complex, dynamic, and bidirectional association between stress and adiposity. The study participants were all women, and these results may not generalize to men. Linking childhood psychosocial conditions with repeated measures of epigenetic age would better disentangle the dynamic process of aging, rather than a single measurement of epigenetic age.^52^ Finally, the aging clocks we studied were assayed using saliva, but they were originally derived in DNA methylation from blood. Although saliva is desirable for studies of community participants, the consistency of interpretation of epigenetic clocks across tissues remains uncertain, suggesting our findings must be replicated in blood measures.

## Conclusions

This cohort study is the first lifespan study, to our knowledge, to demonstrate associations among childhood perceived stress, adiposity, and biological indices of midlife aging and health. This study also highlights the long shadow of childhood SES on adult health, as lower parental educational level was associated with elevated insulin resistance and accelerated epigenetic aging at midlife. Future work can build on these findings to identify malleable factors that may slow the impact of social hallmarks of aging.

## Notes

Supplement 1. **eFigure 1.** Study Timeline **eTable 1.** Comparison of Full, Analytic, and Excluded Samples **eFigure 2.** Correlation Matrix for Key Study Variables **eResults 1.** Moderation by Race **eTable 2.** Synergistic Analysis of Parental Educational Level and Childhood Perceived Stress in Relation to GrimAge **eTable 3.** Synergistic Analysis of Childhood Parental Income and Childhood Perceived Stress in Relation to GrimAge **eTable 4.** Synergistic Analysis of Parental Educational Level and Childhood Perceived Stress in Relation to HOMA-IR **eTable 5.** Synergistic Analysis of Childhood Parental Income and Childhood Perceived Stress in Relation to HOMA-IR **eResults 2.** Additional Mediation Results **eResults 3.** Sensitivity to Outlier Removal **eResults 4.** Intergenerational Educational Experiences and Midlife Insulin Resistance and Epigenetic Age

Supplement 2.
Data Sharing Statement
