# Supplementary material for: Psychosocial Disadvantage During Childhood and Midlife Health: NIMHD Social Epigenomics Program
Source: JAMA Netw Open. 2024 Jul 29;7(7):e2421841. doi: 10.1001/jamanetworkopen.2024.21841 (PMC11287423; doi:10.1001/jamanetworkopen.2024.21841)
Supplement: Supplement 1. — eFigure 1. Study Timeline eTable 1. Comparison of Full, Analytic, and Excluded Samples eFigure 2. Correlation Matrix for Key Study Variables eResults 1. Moderation by Race eTable 2. Synergistic Analysis of Parental Educational Level and Childhood Perceived Stress in Relation to GrimAge eTable 3. Synergistic Analysis of Childhood Parental Income and Childhood Perceived Stress in Relation to GrimAge eTable 4. Synergistic Analysis of Parental Educational Level and Childhood Perceived Stress in Relation to HOMA-IR eTable 5. Synergistic Analysis of Childhood Parental Income and Childhood Perceived Stress in Relation to HOMA-IR eResults 2. Additional Mediation Results eResults 3. Sensitivity to Outlier Removal eResults 4. Intergenerational Educational Experiences and Midlife Insulin Resistance and Epigenetic Age [file jamanetwopen-e2421841-s001.pdf]

Supplementary Online Content

Brown RL, Alegria KE, Hamlat E, et al. Psychosocial disadvantage during childhood and midlife health: NIMHD social epigenomics program. *JAMA Netw Open*. 2024;7(7):e2421841. doi:10.1001/jamanetworkopen.2024.21841

eFigure 1. Study Timeline

eTable 1. Comparison of Full, Analytic, and Excluded Samples

eFigure 2. Correlation Matrix for Key Study Variables

eResults 1. Moderation by Race

eTable 2. Synergistic Analysis of Parental Educational Level and Childhood Perceived Stress in Relation to GrimAge

eTable 3. Synergistic Analysis of Childhood Parental Income and Childhood Perceived Stress in Relation to GrimAge

eTable 4. Synergistic Analysis of Parental Educational Level and Childhood Perceived Stress in Relation to HOMA-IR

eTable 5. Synergistic Analysis of Childhood Parental Income and Childhood Perceived Stress in Relation to HOMA-IR

eResults 2. Additional Mediation Results

eResults 3. Sensitivity to Outlier Removal

eResults 4. Intergenerational Educational Experiences and Midlife Insulin Resistance and Epigenetic Age

This supplementary material has been provided by the authors to give readers additional information about their work.

© 2024 Brown RL et al. *JAMA Network Open*.

eFigure 1. Study Timeline

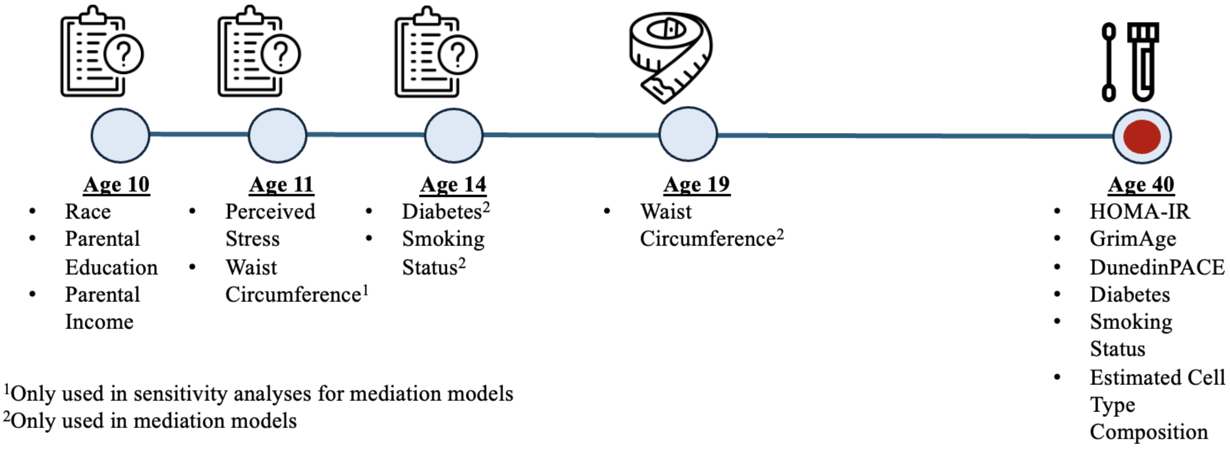

[1]Only used in sensitivity analyses for mediation models
[2]Only used in mediation models

© 2024 Brown RL et al. *JAMA Network Open*.

eTable 1. Comparison of Full, Analytic, and Excluded Samples

Variable		*Full Sample* (*N* = 627[a])	*Analytic Sample* (*n* = 433)	*Excluded Sample* (*n* = 194)	*p*
Age [c]		39.53 (1.28)	39.37 (1.22)	39.88 (1.35)	< .001
Race (%)					.43
	White	51	50	53	
	Black	49	50	47	
Childhood Parental Income (%)					.34
	< $10,000	19	19	19	
	$10,000-$19,999	18	17	21	
	$20,000-$39,999	29	28	31	
	>$40,000	34	36	29	
Parental Education (%)					.44
	High school or less	21	23	18	
	Some college	47	46	48	
	College or more	32	31	34	
Current Smoking Status [c] (%)					.37
	No	81	82	79	
	Yes	19	18	21	
Current Diabetes Status [c] (%)					.20
	No	87	86	90	
	Yes	13	14	10	
Late Adolescent Waist Circumference, cm		76.54 (12.98)	76.31 (12.80)	77.08 (13.40)	.51
Childhood Perceived Stress (Age 11)		25.47 (6.70)	25.69 (6.68)	24.87 (6.74)	.19
Midlife HOMA-IR (Age 40)		3.04 (4.02)	2.93 (3.47)	3.94 (7.17)	.38
Midlife GrimAge2 (Age 40)		61.60 (5.64)	61.60 (5.59)	61.58 (6.18)	.98
Midlife DunedinPACE (Age 40)		1.27 (0.17)	1.28 (0.17)	1.25 (0.56)	.34

Note. Percentages may not sum to 100 due to rounding.

[a] Full sample of participants with midlife follow-up data

[b] *p*-values for difference between analytic and excluded samples; t-tests were used for continuous variables while chi-squared tests were used for categorical variables

© 2024 Brown RL et al. *JAMA Network Open*.

eFigure 2. Correlation Matrix for Key Study Variables

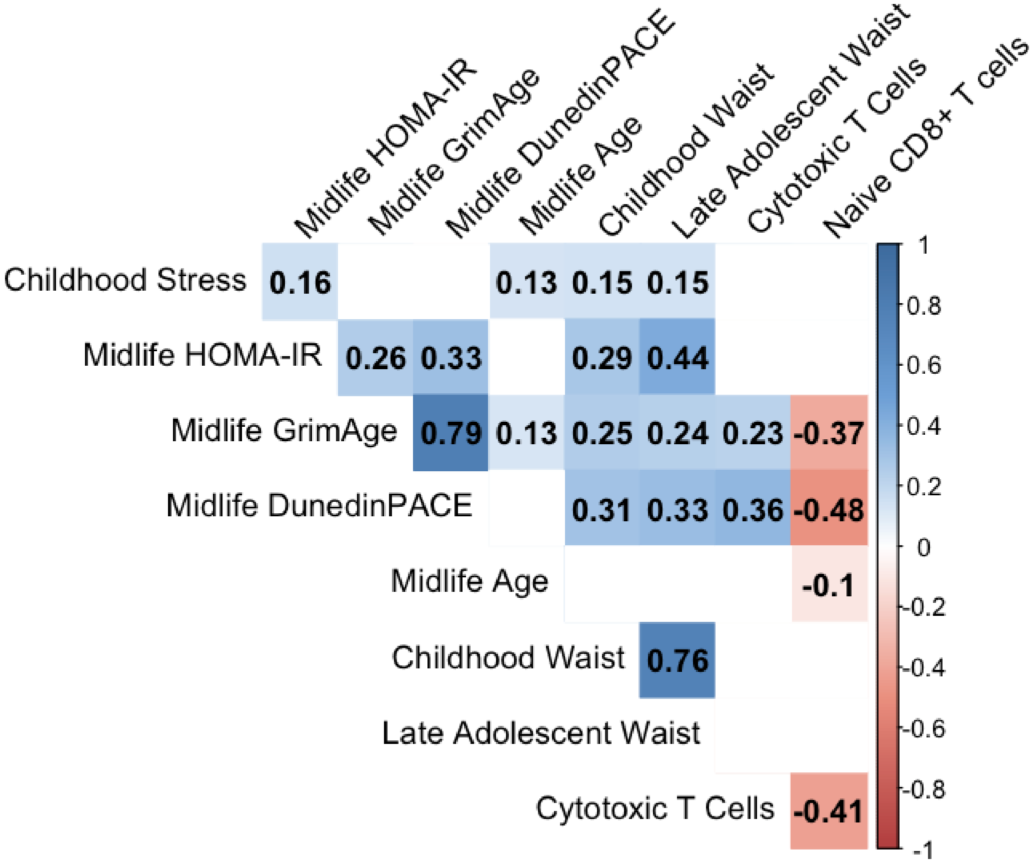

Note. Values presented are correlation coefficients with the direction and strength of association represented visually by color. Non-significant associations are blank, all values here are significant at $p < .05$ level.

© 2024 Brown RL et al. *JAMA Network Open*.

eResults 1. Moderation by Race

Childhood SES

There were no significant interactions between parental education and race when examining HOMA-IR as an outcome, regardless of whether diabetes and smoking status were in the model (*ps* = .14-.34), or not in the model (*ps* = .07-.35). There also were no significant interactions between parental education and race when examining GrimAge as an outcome, regardless of whether diabetes and smoking status were in the model (*ps* = .34-.54), or not in the model (*ps* = .19-.61). There were no significant interactions between parental education and race when examining DunedinPACE as an outcome, regardless of whether diabetes and smoking status were in the model (*ps* = .19-.44), or not in the model (*ps* = .10-.70). In sum, individual associations of parental education with HOMA-IR, GrimAge, and DunedinPACE did not differ based on whether the participant was Black or white.

There were no reliable significant interactions between childhood parental income and race when examining HOMA-IR as an outcome when diabetes and smoking status were not in the model (*ps* = .06-.78). When diabetes and smoking status are included, there was an interaction between childhood parental income and race (b = .51, *p* = .04) such that at the highest parental income level (>$40,000) Black women (estimated marginal mean = 1.18, SE = 0.12) had higher HOMA-IR levels compared to white women (estimated marginal mean = 0.71, SE = 0.09) in this sample (b = -0.47, Tukey HSD-adjusted *p* = .005). No other levels of childhood parental income interacted with race in HOMA-IR models (*ps* = .51-.61). There were no significant interactions between childhood parental income and race when examining GrimAge as an outcome, regardless of whether potential mediators (diabetes, smoking status, estimated cell type proportions) were in the model (*ps* = .21-.99), or not in the model (*ps* = .15-.89). There were also no significant interactions between childhood parental income and race when examining DunedinPACE as an outcome initially (*ps* = .56-.94). After including diabetes, smoking status, and estimated cell type proportions, there was a significant interaction between childhood parental income and race (b = .07, *p* = .04) such that at the highest parental income level (>$40,000) Black women (estimated marginal mean = 1.34, SE = 0.03) had faster DunedinPACE compared to white women (estimated marginal mean = 1.25, SE = 0.03) in this sample (b = -0.09, Tukey HSD-adjusted *p*<.001). In sum, individual associations of childhood parental income with GrimAge did not differ based on whether the participant was Black or white. There was some consistent evidence of an interaction for HOMA-IR and DunedinPACE such that Black women in the highest income group level had higher HOMA-IR and faster DunedinPACE compared to the white women in the highest income group level in this sample.

Childhood Perceived Stress

There were no significant interactions between childhood perceived stress and race when examining HOMA-IR as an outcome, regardless of whether diabetes and smoking status were in the model (b = 0.00, *p* = .90), or not in the model (b = 0.00, *p* = .79). Similarly, there were no significant interactions between childhood perceived stress and race when examining GrimAge as an outcome, regardless of whether diabetes, smoking status, and estimated cell type composition were in the model (b = 0.06, *p* = .28), or not in the model (b = -0.02, *p* = .77). There were also no significant interactions between childhood perceived stress and race when examining DunedinPACE as an outcome, regardless of whether diabetes, smoking status, or estimated cell type composition were in the model (b = 0.00, *p* = .15), or not in the model (b = 0.00, *p* = .77). In sum, individual associations of childhood perceived stress with HOMA-IR, GrimAge, and DunedinPACE did not differ based on whether the participant was Black or white.

© 2024 Brown RL et al. *JAMA Network Open*.

eTable 2. Synergistic Analysis of Parental Educational Level and Childhood Perceived Stress in Relation to GrimAge

	Synergistic Model 1: GrimAge		
Predictors	*std. Beta*	*standardized CI*	*p*
(Intercept)	-0.17	-0.63 – 0.30	0.64
Age	0.07	-0.01 – 0.14	0.07
Diabetes (Yes = 1, No = 0)	0.10	0.03 – 0.17	**0.007**
Current Smoker (Yes = 1, No = 0)	0.92	0.73 – 1.11	**<0.001**
Race (White = 1, Black = 2)	0.44	0.29 – 0.59	**<0.001**
Cluster	-0.02	-0.46 – 0.43	0.94
Cytotoxic T cells	-0.03	-0.11 – 0.06	0.54
Naïve CD8+ T cells	-0.15	-0.23 – -0.06	**0.001**
Naïve CD4+ T cells	0.13	0.05 – 0.20	**0.001**
Granulocytes	0.94	-0.06 – 1.94	0.06
Monocytes	0.48	0.00 – 0.95	**0.05**
B cells	-0.21	-0.48 – 0.05	0.12
CD4+ T cells	0.99	0.42 – 1.55	**0.001**
CD8+ T cells	0.05	-0.08 – 0.17	0.46
Natural Killer cells	0.13	-0.00 – 0.27	0.06
Parental Education: High school or less[a]			
Parental Education: Some college	-0.21	-0.39 – -0.03	0.60
Parental Education: College or more	-0.33	-0.52 – -0.13	0.35
Childhood Perceived Stress	0.04	-0.13 – 0.20	0.66
Parental Education: Some college x Childhood Perceived Stress	-0.00	-0.20 – 0.19	0.97
Parental Education: College or more x Childhood Perceived Stress	0.01	-0.19 – 0.21	0.91

© 2024 Brown RL et al. *JAMA Network Open*.

Observations	391
R^2 / R^2 adjusted	0.568 / 0.546

[a] Reference category

© 2024 Brown RL et al. *JAMA Network Open*.

eTable 3. Synergistic Analysis of Childhood Parental Income and Childhood Perceived Stress in Relation to GrimAge

	**Synergistic Model 2: GrimAge**		
Predictors	*std. Beta*	*standardized CI*	*p*
(Intercept)	-0.26	-0.72 – 0.21	0.734
Age	0.07	0.00 – 0.14	**0.05**
Diabetes (Yes = 1, No = 0)	0.09	0.02 – 0.16	**0.008**
Current Smoker (Yes = 1, No = 0)	0.92	0.73 – 1.10	**<0.001**
Race (White = 1, Black = 2)	0.41	0.26 – 0.56	**<0.001**
Cluster	-0.08	-0.52 – 0.36	0.72
Cytotoxic T cells	-0.02	-0.10 – 0.06	0.69
Naïve CD8+ T cells	-0.13	-0.22 – -0.05	**0.003**
Naïve CD4+ T cells	0.12	0.05 – 0.20	**0.002**
Granulocytes	0.95	-0.03 – 1.93	0.06
Monocytes	0.45	-0.01 – 0.92	0.05
B cells	-0.20	-0.47 – 0.06	0.13
CD4+ T cells	1.02	0.46 – 1.57	**<0.001**
CD8+ T cells	0.05	-0.07 – 0.17	0.40
Natural Killer cells	0.14	0.01 – 0.28	**0.03**
Childhood Parental Income: < $10,000[a]			
Childhood Parental Income: $10,000-$19,999	0.23	0.01 – 0.45	**0.003**
Childhood Parental Income: $20,000-$39,999	-0.14	-0.35 – 0.06	0.72
Childhood Parental Income: >$40,000	-0.10	-0.31 – 0.11	0.68
Childhood Perceived Stress	0.07	-0.09 – 0.24	0.34
Childhood Parental Income: $10,000-$19,999 x Childhood Perceived Stress	-0.31	-0.54 – -0.07	**0.01**

© 2024 Brown RL et al. *JAMA Network Open*.

Predictors	*std. Beta*	*standardized CI*	*p*
Childhood Parental Income: $20,000-$39,999 x Childhood Perceived Stress	0.00	-0.20 – 0.20	.99
Childhood Parental Income: >$40,000 x Childhood Perceived Stress	0.02	-0.18 – 0.22	0.86
Observations	391		
R^2 / R^2 adjusted	0.586 / 0.562		

[a] Reference category

© 2024 Brown RL et al. *JAMA Network Open*.

eTable 4. Synergistic Analysis of Parental Educational Level and Childhood Perceived Stress in Relation to HOMA-IR

	Synergistic Model 1: HOMA-IR		
Predictors	*std. Beta*	*standardized CI*	*p*
(Intercept)	-0.06	-0.33 – 0.20	0.30
Age	-0.02	-0.12 – 0.08	0.66
Diabetes (Yes = 1, No = 0)	0.78	0.45 – 1.10	**<0.001**
Current Smoker (Yes = 1, No = 0)	-0.02	-0.29 – 0.25	0.88
Race (White = 1, Black = 2)	0.34	0.12 – 0.55	**0.002**
Parental Education: High school or less[a]			
Parental Education: Some college	-0.17	-0.44 – 0.09	0.05
Parental Education: College or more	-0.33	-0.63 – -0.04	0.08
Childhood Perceived Stress	-0.04	-0.30 – 0.22	0.75
Parental Education: Some college x Childhood Perceived Stress	0.25	-0.05 – 0.55	0.10
Parental Education: College or more x Childhood Perceived Stress	0.19	-0.12 – 0.49	0.23
Observations	336		
R^2 / R^2 adjusted	0.156 / 0.133		

[a] Reference category

© 2024 Brown RL et al. *JAMA Network Open*.

eTable 5. Synergistic Analysis of Childhood Parental Income and Childhood Perceived Stress in Relation to HOMA-IR

	Synergistic Model 2: HOMA-IR		
Predictors	*std. Beta*	*standardized CI*	*p*
(Intercept)	-0.22	-0.53 – 0.10	0.61
Age	-0.02	-0.13 – 0.08	0.64
Diabetes (Yes = 1, No = 0)	0.79	0.46 – 1.12	**<0.001**
Current Smoker (Yes = 1, No = 0)	0.01	-0.26 – 0.28	0.94
Race (White = 1, Black = 2)	0.35	0.13 – 0.57	**0.002**
Childhood Parental Income: < $10,000[a]			
Childhood Parental Income: $10,000-$19,999	0.10	-0.25 – 0.46	0.25
Childhood Parental Income: $20,000-$39,999	-0.12	-0.44 – 0.20	0.58
Childhood Parental Income: >$40,000	-0.05	-0.37 – 0.27	0.66
Childhood Perceived Stress	0.18	-0.10 – 0.46	0.21
Childhood Parental Income: $10,000-$19,999 x Childhood Perceived Stress	-0.20	-0.57 – 0.17	0.29
Childhood Parental Income: $20,000-$39,999 x Childhood Perceived Stress	0.07	-0.27 – 0.40	0.70
Childhood Parental Income: >$40,000 x Childhood Perceived Stress	-0.09	-0.41 – 0.23	0.58
Observations	336		
R^2 / R^2 adjusted	0.148 / 0.120		

[a] Reference category

© 2024 Brown RL et al. *JAMA Network Open*.

eResults 2. Additional Mediation Results

Sensitivity Analyses for Primary Mediations

In a sensitivity analysis, we tested the robustness of our primary mediation findings by additionally covarying for waist circumference at age 11 in Path A. This analysis examines whether childhood perceived stress is associated with the *change* in adiposity from age 11 to age 19. For HOMA-IR (*n* = 302), the indirect association of childhood perceived stress through late adolescent adiposity on midlife HOMA-IR remained significant, b=0.002, 95% CI [0.0001,0.00], *p*=.04, though this should be interpreted with caution as the confidence interval includes zero indicating that the true effect may not be different from zero. Regarding GrimAge (*n* = 345), childhood perceived stress was no longer indirectly related to midlife GrimAge through late adolescent adiposity, b=0.007, 95% CI [-0.001,0.02], *p*=.06.

Non-Significant SES Mediations

The association between education and HOMA-IR was not significantly mediated by late adolescent adiposity, b=-0.03, 95% CI [-0.06,0.00], *p*=.08, nor was the association between income and HOMA-IR, b=-0.02, 95% CI [-0.06,0.00], *p*=.09. Similarly, the association between education and GrimAge was not significantly mediated by late adolescent adiposity, b=-0.02, 95% CI [-0.16,0.09], *p*=.68, nor was the association between income and GrimAge, b=0.03, 95% CI [-0.30,0.35], *p*=.77.

© 2024 Brown RL et al. *JAMA Network Open*.

eResults 3. Sensitivity to Outlier Removal

Thirteen individuals were excluded from HOMA-IR analyses due to large (i.e., +2SD) HOMA-IR values. In this section, we show key HOMA-IR analyses with these individuals included to highlight that our pattern of results is unchanged based on exclusion of these individuals.

Sensitivity Analysis of Additive Associations

When examining additive associations with HOMA-IR outliers included, results are largely similar with the exception of one additional level of education being associated with HOMA-IR. Specifically, there was evidence that having a parent with some college (b=0.20, SE=0.10, *p*=.04) or college+ (b=0.25, SE=0.11, *p*=.02) was associated with lower HOMA-IR compared to those whose parents had a high school degree or less. Simultaneously, higher childhood perceived stress was also associated with higher HOMA-IR (b=0.01, SE=0.01, *p*=.02).

Sensitivity Analysis for Mediation Analyses

When examining mediation analyses with HOMA-IR outliers included (*n* = 314), results follow the same pattern as when these individuals are excluded. In Path A, childhood perceived stress was associated with higher adiposity in late adolescence, b=0.34, SE=0.11, *p*=.002; in turn, in Path B, higher late adolescent adiposity was associated with elevated midlife HOMA-IR, b=0.020, SE=0.003, *p*<.001. The total association of childhood perceived stress on HOMA-IR at age 40 was not significant (b= 0.01, 95% CI [-0.001,0.02], *p*=.09), nor was the direct association (b=0.01, 95% CI [-0.008,0.02], *p*=.44). Instead, childhood perceived stress was statistically significantly associated through adiposity in late adolescence with midlife HOMA-IR, b=0.01, 95% CI [0.002,0.01], *p*<.001.

Next, we included childhood waist as a covariate in Path A (i.e., shifting the question to focus on *change* in adiposity from age 11 to age 19 as a mediator; *n* = 313). The indirect association of childhood perceived stress through late adolescent adiposity on midlife HOMA-IR was no longer significant, b=0.003, 95% CI [-0.00,0.01], *p*=.07.

© 2024 Brown RL et al. *JAMA Network Open*.

eResults 4. Intergenerational Educational Experiences and Midlife Insulin Resistance and Epigenetic Age

Exploratory post-hoc analyses tested competing models regarding the relationship between parental education and one's own education related to midlife insulin resistance and epigenetic age. We examined: (1) moderation models whereby the association between parental education and the outcomes may depend on one's own level of education and (2) mediation models whereby the association between parental education and the outcomes may be explained indirectly through one's own level of education.

There was no evidence for an interaction between parental education and one's own education associated with HOMA-IR (*p*s: .62-.85), GrimAge (*p*s: .62-.85), nor DunedinPACE (*p*s: .34-.94) (see Supplementary Material for full results). However, for GrimAge, we saw evidence for mediation such that in Path A, higher childhood parental education was associated with one's own higher education, b=0.26, SE=0.05, $p<.001$; in turn, in Path B, one's own higher educational attainment was associated with lower midlife GrimAge, b=-0.84 years, SE=0.31, $p=.006$. Thus, parental education was indirectly statistically significantly associated with GrimAge through one's own level of education (n = 384; b=-0.22, 95% CI [-0.46,-0.04], $p=.006$, after covarying for age, adiposity, race, diabetes status, smoking status, cluster, estimated cell type composition variables, and parental income. We did not observe the same pattern for HOMA-IR or DunedinPACE. In sum, these analyses highlight that parental education may affect offspring epigenetic aging through enhanced higher educational opportunities for those whose parents had greater educational opportunity.

© 2024 Brown RL et al. *JAMA Network Open*.
