# Supplementary material for: Psychosocial Disadvantage During Childhood and Midlife Health: NIMHD Social Epigenomics Program
Source: JAMA Netw Open. 2024 Jul 29;7(7):e2421841. doi: 10.1001/jamanetworkopen.2024.21841 (PMC11287423; doi:10.1001/jamanetworkopen.2024.21841)
Supplement: Supplement 2. — Data Sharing Statement [file jamanetwopen-e2421841-s002.pdf]

Data Sharing Statement

Brown. Psychosocial Disadvantage During Childhood and Midlife Health. *JAMA Netw Open*. Published July 29, 2024. doi:10.1001/jamanetworkopen.2024.21841

Data
Data available: Yes
Data types: Deidentified participant data, Data dictionary
How to access data: ryan.l.brown@ttu.edu
When available: With publication

Supporting Documents
Document types: Statistical/analytic code
How to access documents: ryan.l.brown@ttu.edu
When available: With publication

Additional Information
Who can access the data: Researchers whose proposed use of the data has been approved by the NGHS study team.
Types of analyses: For any reasonable purpose.
Mechanisms of data availability: After approval of a proposal or written statement of intent.
